# Role of miRs in immune suppression

**DOI:** 10.1186/2051-1426-2-S3-P268

**Published:** 2014-11-06

**Authors:** Barbara Seliger, Simon Jasinski-Bergner, Evamaria Gonschorek, Marian Teren, Christine Stoehr, Anne Meinhardt, Franziska Stehle, Kristin Schulz, Bernd Wullich, Arndt Hartmann

**Affiliations:** 1 Institute of Medical Immunology, Germany; 2 Martin Luther University Halle-Wittenberg, Germany; 3 Institute of Medical Immunology, Halle, Germany; 4 Institute of Pathology, University of Erlangen, Germany; 5 Clinic of Urology, University Hospital Erlangen, Erlangen, Germany; 6 Institute of pathology, University Hospital Erlangen, Erlangen, Germany

## 

Tumors evade adaptive and/or innate immune response multiple mechanisms including loss and/or down-regulation of HLA class I antigens due to reduced or impaired expression of single or multiple components of the antigen processing machinery (APM), loss of co-stimulatory molecules, expression of co-inhibitory molecules including B7-H family members as well as of the non-classical HLA-G antigen. This could lead to a reduced immune response mediated by various immune cell subpopulations. Due to the identification of microRNAs (miRs) that play a key role in tumor formation and/or suppression and in modulating immune cell differentiation as well as adaptive and innate immune responses the complexity of the molecular mechanisms of immune escape of tumors has increased. Therefore, the aim of the study is to determine the impact of miRs on tumor immune surveillance by identification of miRs modulating the expression of single or multiple APM components, B7-H1 molecules and/or HLA-G using different experimental approaches. *In silico *analysis, miR arrays, deep sequencing, luciferase reporter assays as well as enrichment of miRs by miTRAP led to the identification of a number of miRs involved in the different immune escape mechanisms. These distinct miRs identified functionally regulate the expression of the respective immune modulatory molecules, which directly affect the immune recognition of tumor cells. Furthermore, combining immunohistochemistry with miR expression using a tissue microarray revealed an inverse relationship of miR and HLA-G expression, which was associated with an increased tumor grading, but not with an enhanced tumor patients' survival. Furthermore, overexpression of these immune modulatory miRs results in altered protein expression pattern. In particular a high frequency of differentially expressed proteins involved in the cellular metabolism, cell proliferation, apoptosis and gene transcription could be identified suggesting that miRs exert a dual role by modulating both the tumorigenicity as well as the immunogenicity of tumor cells. Thus, these miRs might provide novel therapeutic targets and/or might be used as diagnostic or prognostic tools in cancers.

